# Iron complexes of tetramine ligands catalyse allylic hydroxyamination via a nitroso–ene mechanism

**DOI:** 10.3762/bjoc.11.275

**Published:** 2015-12-11

**Authors:** David Porter, Belinda M-L Poon, Peter J Rutledge

**Affiliations:** 1 School of Chemistry F11, The University of Sydney, NSW 2006, Australia

**Keywords:** CH activation, hydroxyamination, iron catalysis, nitroso-ene

## Abstract

Iron(II) complexes of the tetradentate amines tris(2-pyridylmethyl)amine (TPA) and *N*,*N*′-bis(2-pyridylmethyl)-*N*,*N*′-dimethylethane-1,2-diamine (BPMEN) are established catalysts of C–O bond formation, oxidising hydrocarbon substrates via hydroxylation, epoxidation and dihydroxylation pathways. Herein we report the capacity of these catalysts to promote C–N bond formation, via allylic amination of alkenes. The combination of *N*-Boc-hydroxylamine with either FeTPA (1 mol %) or FeBPMEN (10 mol %) converts cyclohexene to the allylic hydroxylamine (*tert*-butyl cyclohex-2-en-1-yl(hydroxy)carbamate) in moderate yields. Spectroscopic studies and trapping experiments suggest the reaction proceeds via a nitroso–ene mechanism, with involvement of a free *N*-Boc-nitroso intermediate. Asymmetric induction is not observed using the chiral tetramine ligand (+)-(2*R*,2′*R*)-1,1′-bis(2-pyridylmethyl)-2,2′-bipyrrolidine ((*R*,*R*′)-PDP).

## Introduction

The selective functionalization of C–H bonds is an area of considerable current research interest [1–5]. The development of methods for catalytic C–H amination has attracted particular attention [6–11], given the significance of C–N bonds to the structures of biologically active natural products and pharmaceuticals. In this context there has been a renewed focus on the chemistry of acylnitroso species in recent times [12–15], in particular on α-hydroxyamination of carbonyl compounds via nitrosocarbonyl aldol reactions [16–21] and allylic hydroxyamination of alkenes via nitroso–ene reactions [22–26]. Several new developments in the related hetero-Diels–Alder reaction of acylnitroso species have also been reported recently [27–30].

These methodologies generally involve in situ generation of the acylnitroso species, achieved using a variety of oxidants including vanadium- [28], manganese- [19–21], iron- [23–24], copper- [22,31], rhenium- [26], and rhodium- [27] based reagents.

The recent resurgence of interest in the nitroso–ene reaction builds on earlier work by Sharpless, Nicolas, Jørgensen and others. Sharpless reported allylic amination of 2-methyl-2-hexene with *N*-(*p*-chlorophenyl)hydroxylamine using a molybdenum complex [32], a process that was made catalytic by adding excess *N*-phenylhydroxylamine [33]. The combination of iron(II) phthalocyanines [34–35] or iron(II)/iron(III) chloride [36–38] and *N*-phenylhydroxylamine effect allylic amination reactions that are believed to follow a nitroso–ene mechanism. Similar reactions have been reported using copper salts and *N*-phenylhydroxylamine [39] or *N*-Boc-hydroxylamine [40–41], presumably via oxidation of the hydroxylamine to a nitroso species which then undergoes the nitroso–ene reaction.

**Figure 1:**
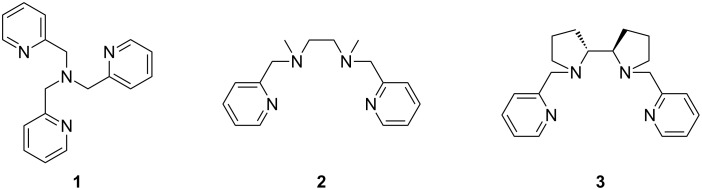
TPA (**1**), BPMEN (**2**) and (*R*,*R*′)-PDP (**3**) ligands.

Stemming from our interest in iron-catalysed hydrocarbon oxidation using systems inspired by the non-heme iron-dependent enzyme family [42–47], we have investigated the capacity of iron complexes of simple tetramine ligands to promote the reaction between an alkene and *N*-Boc-hydroxylamine. Herein we report that iron complexes of tris(2-pyridylmethyl)amine (TPA, **1**) [48–49], *N*,*N*′-bis(2-pyridylmethyl)-*N*,*N*′-dimethylethane-1,2-diamine (BPMEN, **2**) [48,50] and (+)-(2*R*,2′*R*)-1,1′-bis(2-pyridylmethyl)-2,2′-bipyrrolidine ((*R*,*R*′)-PDP, **3**) [51] (Figure 1) catalyse the allylic amination of cyclohexene. Mechanistic investigations suggest the reaction proceeds via nitroso–ene reaction of the oxidised hydroxylamine and the alkene.

## Results and Discussion

### Synthesis of metal complexes

The tetramine ligands TPA (**1**), BPMEN (**2**) and (*R*,*R*′)-PDP (**3**) were synthesised following literature procedures [48,50–51], then combined with iron(II) triflate as previously reported to generate the complexes [Fe(TPA)(CH_3_CN)_2_](OTf)_2_ (FeTPA, **4**) [52], [Fe(BPMEN)(OTf)_2_] (FeBPMEN, **5**) [48] and [Fe(*R*,*R*′-PDP)(OTf)_2_] (Fe(*R*,*R*′)-PDP, **6**) [51].

### Allylic amination reactions

**Scheme 1:**
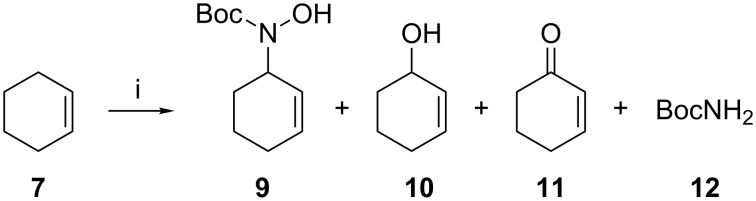
Allylic hydroxyamination of cyclohexene (**7**) using iron catalysts **4** and **5**; i. **4** or **5** (10 mol %), BocNHOH (**8**), CH_3_CN, rt, 18 h; for yields see Table 1.

As an extension of our previously reported iron-catalysed allylic oxidation of cyclohexene (**7**) [45–47], we wished to explore potential C–N bond formation at this position using iron catalysis. Combining cyclohexene (**7**, in excess) with *N*-Boc-hydroxylamine (**8**) as the nitrogen source and the iron complex FeTPA (**4**) or FeBPMEN (**5**) afforded a mixture of products: the allylic hydroxylamine **9** alongside the Fenton oxidation products alcohol **10** and ketone **11** [53], and a small amount of *tert-*butyl carbamate (**12**, Scheme 1). Initial reactions under an argon or air atmosphere returned product mixtures in the ratios shown in Table 1.

**Table 1:** Catalytic allylic amination of cyclohexene (Scheme 1). Reaction conditions: catalyst **4** or **5** (7 μmol) and cyclohexene (0.7 mL, 7 mmol) were dissolved in CH_3_CN (total volume 10 mL) and stirred at room temperature under air or argon atmosphere while BocNHOH (**8**, 70 μmol) was added, then stirring was continued overnight (18 h).

Entry^a^	Catalyst	mol %	Atmosphere	**9**^b,c^	**10**^b,c^	**11**^b,c^	**12**^b,c^

1	**4**	10	argon	10	5	2	2
2	**4**	10	air	27	54	36	5
3	**5**	10	argon	9	6	2	0
4	**5**	10	air	40	32	14	4

^a^Each reaction was performed in triplicate; data are averages of at least three runs. ^b^Yields determined by GC using single point internal standard method (Tables S1 and S2, Supporting Information File 1). ^c^Yields are quoted relative to the initial amount of BocNHOH (**8**), limiting reagent for the hydroxyamination reaction of interest. Formation of products **10** and **11** is not dependent on hydroxylamine **8** so the combined yields for some entries in this table are more than 100%.

Under an argon atmosphere, the allylic hydroxylamine **9** was produced in ~10% yield with either ligand; performing the reaction open to air lifted the yield of the allylic hydroxyamination product **9** as high as 40%, but also substantially increased yields of **10** and **11** (Table 1).

Control experiments using just the metal salt or each of the ligands on their own returned trace quantities of product **9** and varying levels of Fenton-type pathways (Table S3, Supporting Information File 1), confirming that FeTPA (**4**) and FeBPMEN (**5**) are active agents in promoting allylic hydroxyamination of cyclohexene.

The effect of catalyst loading was screened under an air atmosphere, since initial results indicated that better yields of **9** are obtained under air than argon. Thus cyclohexene (0.7 mL, 7 mmol, 100 equiv) was added to a solution of catalyst **4** or **5** (1–20 mol %) and BocNHOH (70 μmol, 1 equiv) in CH_3_CN (Table S4, Supporting Information File 1). Lowering the catalyst loading of FeTPA from 10 to 5 mol % led to a small increase in the yield of allylic hydroxylamine **9** with a significant decrease in the appearance of allylic oxidation products **10** and **11**. The amount of FeTPA (**4**) could be further lowered to 2 and 1 mol %, bringing further small increases in the yield of **9**. However increasing loading of FeTPA (**4**) to 20 mol % halts the amination reaction, returning only allylic oxidation products **10** and **11**. In contrast, changing the catalyst loading of FeBPMEN (**5**) up or down from 10 mol % lowers yields of **9**; at 1 mol % or 20 mol % loading of catalyst **5**, increased levels of **10** and **11** are observed, but at 5 mol % catalyst **5**, the yields of all three oxidation products are diminished. Clearly the competing hydroxyamination and Fenton reaction pathways are sensitive to the amount of catalyst used relative to BocNHOH; optimum catalyst loadings are 1 mol % for **4** and 10 mol % for **5**.

Nicholas and Kalita have reported that the addition of hydrogen peroxide can improve yields in their copper-catalysed allylic amination reactions using BocNHOH [41]. Thus the addition of hydrogen peroxide (1:1 relative to BocNHOH) to reactions with **4** or **5** was investigated. Using a 1:1:1 ratio of cyclohexene:BocNHOH:H_2_O_2_ with FeTPA (**4**) at 1 mol %, allylic hydroxylamine **9** was formed in only 4% yield, with the allylic oxidation products **9** and **10** predominant. This is not unexpected given the propensity of hydrogen peroxide to react directly with iron complexes to produce **10** and **11** via Fenton-type pathways [47,53].

We have previously observed solvent-dependent behaviour by non-heme iron complexes when mediating oxidation of cyclohexene in methanol versus acetonitrile as solvent [46–47]. Using methanol as solvent in the allylic amination reactions with FeTPA (**4**, 1 mol %) and cyclohexene in excess, yields of allylic hydroxylamine **9** dropped: **9** was formed in 10% yield (vs 27% in acetonitrile), while yields of allylic oxidation products **10** and **11** were also lowered, to 25% and 38% respectively (vs 54% and 36% in acetonitrile). BocNH_2_ (**12**) was not observed. Using FeBPMEN (**5,** 10 mol %) in methanol, **10** and **11** were formed but target compound **9** was not observed at all. Presumably with methanol as solvent, the oxidising power of iron:ligand system is partially redirected to oxidise the solvent.

With a view to improving the synthetic potential of this reaction, the transformation was attempted at a 1:1 ratio of BocNHOH to cyclohexene. Thus BocNHOH (70 μmol), FeTPA **4** (1 mol %) and cyclohexene (70 μmol) were combined in acetonitrile and stirred for 18 hours at room temperature, open to the air. Allylic hydroxylamine **9** was formed in 6% yield; allylic oxidation products **10** and **11** were each observed in ≤1%. Reaction at 2:1 BocNHOH:cyclohexene did not significantly improve the yield of allylic amine **9** (7%). Similar results were obtained using FeBPMEN (**5**, 10 mol %) as catalyst, which yielded small amounts of **9** (8%) and **10** (2%) but not ketone **11**. In their work using copper(I) iodide to catalyse similar reactions, Iwasa et al. conducted reactions at much higher concentrations of hydroxylamine and alkene (0.5 mmol BocNHOH and 0.75 mmol alkene in a total reaction volume of 1 mL) [40]. With this in mind, the FeBPMEN (**5**) reaction was repeated at 10-fold higher concentration (i.e., 1:1 BocNHOH/cyclohexene in a total reaction volume of 1 mL). Under these conditions the yield of allylic amine **9** doubled relative to the more dilute 1:1 reaction, to 17%; **10** and **11** were not observed.

### Reaction using a chiral catalyst

The chiral catalyst Fe(*R*,*R*′)-PDP (**6**) has been used previously to promote asymmetric C–H oxidation reactions [51]. With a view to developing an asymmetric iron-catalysed allylic hydroxyamination reaction, catalyst **6** was prepared and used to effect conversion of cyclohexene **7** to hydroxylamine **9**. This reaction afforded **9** in 13% yield, but only as the racemate: analysis by chiral GC (CP-Chirasil-Dex CB column) revealed two peaks with equal peak areas (*t*_R_ = 8.6 and 8.8 minutes); the same peaks in the same ratio were observed using a reference sample of racemic **9**.

Several groups have recently reported efficient methods for the asymmetric hydroxyamination of carbonyl compounds using acylnitroso species generated in situ along with chiral oxazolines [16–17], *N*-oxides [19] or amines [18,20–21] as ligands or organocatalysts. In these nitrosoaldol contexts, the chiral agents induce asymmetry by virtue of their influence over the enolate reaction partner. Achieving asymmetric induction in the nitroso–ene reaction is a trickier proposition [14], although this has been demonstrated in an intramolecular context [22].

### Mechanistic studies

**Scheme 2:**
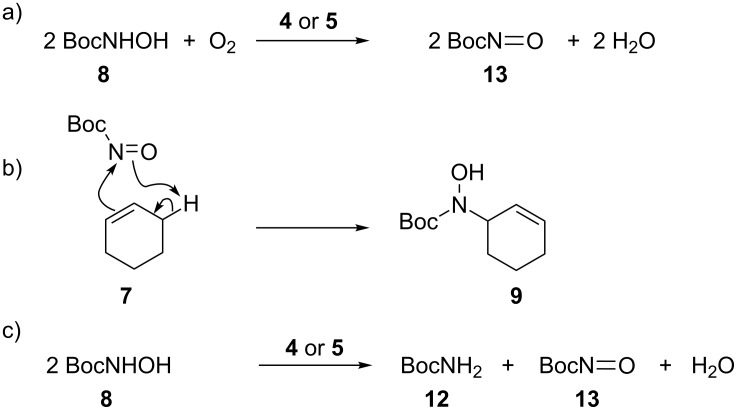
Proposed mechanism for hydroxyamination of cyclohexene (**7**) by FeTPA (**4**) and FeBPMEN (**5**): (a) iron-mediated oxidation of BocNHOH (**8**) by O_2_ affords the nitroso-species **13**, which (b) undergoes an ene reaction with the alkene substrate; (c) an alternative disproportionation reaction to convert **8** to **13** can occur without involvement of O_2_ and also generates BocNH_2_ (**12**), observed as a byproduct at low levels (Tables 1, S3 and S4, Supporting Information File 1). The mechanistic evidence gathered to date suggests the formation of a free nitroso species and ‘off-metal’ reaction with the alkene.

Previous studies of iron-promoted allylic amination reactions with *N*-phenylhydroxylamine, and copper-catalysed reactions with *N*-Boc-hydroxylamine (**8**) return regio- and chemoselectivity profiles that are consistent with reaction via nitroso–ene mechanisms [35–36,38,41]. Thus we hypothesised that the hydroxyamination reactions mediated by FeTPA (**4**) and FeBPMEN (**5**) follow a similar mechanism: iron-catalysed oxidation of **8** to generate the *N*-Boc-nitroso intermediate **13**, which then participates in an ene reaction with cyclohexene (**7**, Scheme 2). Several experiments were conducted to test this hypothesis.

Spectroscopic experiments evince interactions between *N*-Boc-hydroxylamine (**8**) and FeTPA (**4**) in solution. An acetonitrile solution of FeTPA (**4**) is a dark red-brown colour; adding BocNHOH prompts a colour change to deep purple (λ_max_ = 550 nm, ε ≈ 300 L mol^−1^ cm^−1^). Monitoring the electronic spectrum between 400 and 900 nm (Figure S1, Supporting Information File 1), this absorption peak reaches a maximum when 1 equivalent of *N*-Boc-hydroxylamine (**8**) has been added, consistent with coordination of hydroxylamine **8** to the FeTPA complex (**4**). A similar result is observed using ^1^H NMR: titrating a solution of hydroxylamine **8** in *d*_3_-acetonitrile with FeTPA (**4**) indicates coordination of hydroxylamine **8** to the complex **4** (Figure S2, Supporting Information File 1).

Direct observation of the proposed Boc-nitroso intermediate **13** is difficult given its reactive nature. However BocNH_2_ (**12**), the other product of the proposed disproportionation of *N*-Boc-hydroxylamine (**8**, Scheme 2c) is observed as a side product. When *N*-Boc-hydroxylamine (**8**) was treated with FeTPA (**4**) in the absence of an alkene partner, BocNH_2_ (**12**) was isolated in increased yield (25%). This mirrors the results of Jørgensen and Nicholas who have observed an analogous iron-catalysed reduction of *N*-phenylhydroxylamine to aniline, and confirms that FeTPA (**4**) can mediate the conversion of BocNHOH to BocNH_2_.

**Scheme 3:**
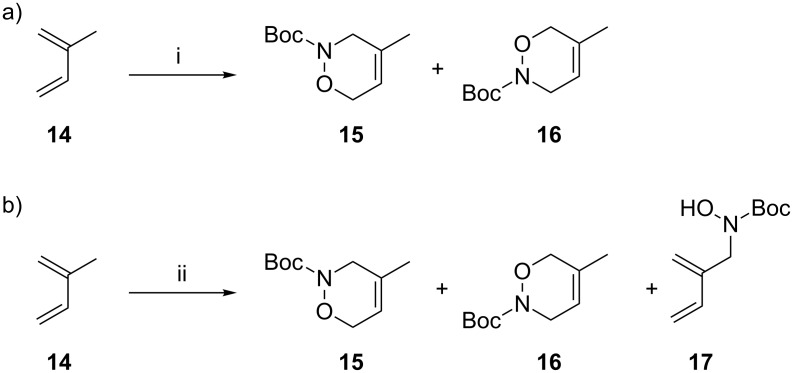
Reaction of isoprene (**14**) under (a) Kirby’s conditions [54–55] and (b) FeTPA- or FeBPMEN-mediated hydoxyamination conditions, which afford the hetero-Diels–Alder products **15** and **16** alongside the hydroxyamination product **17**. Reaction conditions: i. NaIO_4_, BocNHOH (**8**), NaOAc buffer (0.5 mol L^−1^, pH 6), H_2_O/EtOAc, 0 °C, 1 h, product ratio **15**:**16** is 1:1 (by GC); ii. **4** or **5** (10 mol %), BocNHOH (**8**), CH_3_CN, rt, 18 h, product ratio **15**:**16**:**17** is 1:1:3 (by GC).

Nitroso species can be trapped by hetero-Diels–Alder reaction with dienes [54–55], and detection of the resulting hetero-Diels–Alder adducts used to confirm the formation of free nitroso intermediates. Thus trapping experiments were conducted using isoprene (**14**) to investigate the formation of a nitroso species in this reaction. First a 1:1 mixture of cycloadducts **15** and **16** was synthesised as a reference sample using Kirby’s conditions for the hetero-Diels–Alder reaction (*N*-Boc-hydroxylamine (**8**), isoprene (**14**) and sodium periodate, Scheme 3a) [54–55]. Then isoprene (**14**) and *N*-Boc-hydroxylamine (**8**) were combined in acetonitrile in the presence of FeTPA (**4**) or FeBPMEN (**5**). Cycloadducts **15** and **16** were formed, along with the allylic amination product **17** in a 1:1:3 ratio (Scheme 3b); the observation of **15** and **16** in this reaction confirms that a Boc-nitroso species **13** is formed in the FeTPA/FeBPMEN-catalysed reaction. It is interesting to note that the nitroso–ene product is not generally observed under the Kirby conditions, as the allylic hydroxyamination product **17** is unstable in the highly oxidising environment rendered by sodium periodate [56]. Observation of this product in the FeTPA- and FeBPMEN-mediated reaction of isoprene indicates the relative mildness of these conditions for nitroso formation.

Nicholas et al. have reported a similar experiment to study the iron(II,III) chloride-catalysed reaction of *N*-phenylhydroxylamine with 2,3-dimethyl-1,3-butadiene, in which the Diels–Alder cylcoadduct was not observed, and only the allylic amination product was formed [36,38]. Conversely Jørgensen and Johannsen report that *N*-phenylhydroxylamine and 1,3-cyclohexadiene in the presence of iron(II) phthalocyanine do form the Diels–Alder cycloadduct [35]. Although the outcomes with the different catalysts were contrasting, only one product was observed in each case. Cenini et al. have reported that both cycloadduct and ene product are formed in the ruthenium-catalysed reaction of nitrobenzene (an alternate route to a nitrosobenzene intermediate) with isoprene [57]. As mentioned above, isoprene produces two different regioisomers in the hetero-Diels–Alder reaction with nitroso compounds, yet Cenini et al. only detected one isomer. From this observation they concluded that their Ru-catalysed amination reaction and the Diels–Alder reactions were occurring ‘on metal’ without generation of a free nitroso species [57].

In contrast, the FeTPA-mediated reaction generates both BocNO cycloadducts of isoprene along with the amination product. Furthermore, the reaction with the chiral system **6** renders zero asymmetric induction. Thus we conclude that the Fe-TPA/BPMEN reaction involves the nitroso–ene reaction of a free nitroso intermediate.

## Conclusion

FeTPA (**4**) and FeBPMEN (**5**) are established catalysts for the hydroxylation, dihydroxylation and epoxidation of hydrocarbon substrates [48,58–60]. In this study we have shown that they can also catalyse the allylic hydroxyamination of alkenes with *N*-Boc-hydroxylamine. Mechanistic investigations suggest the involvement of a free nitroso species which undergoes a nitroso–ene reaction with the alkene. The intermediacy of a free nitroso species means that asymmetric induction is not observed in reactions with the chiral catalyst Fe(*R*,*R*′)-PDP (**6**).

## Experimental

### General experimental

All commercially available reagents were used without purification unless otherwise specified. Solvents for extraction and chromatography were distilled before use. Solvents for reactions were freshly distilled immediately prior to use. Tetrahydrofuran (THF) was dried over sodium wire and benzophenone. Dichloromethane and acetonitrile were dried over calcium hydride. Acetonitrile was degassed using three freeze–thaw cycles when it was to be used in an atmosphere of argon. Methanol (MeOH) was dried over magnesium methoxide. Alkenes used in allylic amination reactions were passed through a micro-column of neutral alumina immediately before use. Water was purified using a Millipore purification system. Analytical thin-layer chromatography (TLC) was performed using preconditioned plates (Merck Kieselgel 60 F254). Developed TLC plates were viewed using a UV lamp at a wavelength of 254 nm and visualised with a ninhydrin stain. Flash column chromatography was performed on Davisil Grace Davison 40–63 μm (230–400 mesh) silica gel using distilled solvents.

Melting points were recorded on a Stanford Research Systems Optimelt automated melting system and are uncorrected. ^1^H NMR spectra were recorded on Bruker Avance DPX 200 and DPX 300 spectrometers. Chemical shifts are reported in ppm relative to tetramethylsilane or residual solvent resonance as an internal standard. Spectra are reported as signal (ppm), relative integral and multiplicity (singlet s, doublet d, triplet t, doublet of doublets dd, doublet of triplets dt, apparent app, multiplet m, broad br). Coupling constants *J* are reported in Hz to the nearest 0.5 Hz. ^13^C NMR spectra were recorded on a Bruker Avance DPX 300 (75.5 MHz) spectrometer. Chemical shifts are reported in ppm using the residual solvent resonance as an internal standard. Spectra were assigned using DEPT editing where necessary. Low resolution mass spectra were recorded on a Finnigan LCQ MS Detector (ESI, APCI) by Dr. Keith Fisher. Optical rotations were recorded on a Perkin-Elmer Model 341 Polarimeter at 20 °C with a sodium lamp (589 nm), and are reported as 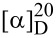 (*c* mg mL^−1^, solvent). Infrared spectra were recorded on a Bruker ALPHA FTIR spectrophotometer (ZnSe ATR). Gas chromatography was carried out on a Hewlett Packard 5890A and 5890 Series II Gas chromatographs with ChemStation software using HP1 (Crosslinked Methyl Silicone Gum) and CP-Chirasil-Dex CB columns, respectively. Both chromatographs were equipped with split/splitless capillary inlets and flame ionization detectors (FID). UV–vis spectra were recorded on a Varian Carey 4000 UV–vis spectrophotometer.

### Synthesis of iron complexes **4**, **5** and **6** and *N*-Boc-hydroxylamine (**8**)

Tris(2-pyridylmethyl)amine (TPA, **1**) [48] and *N*,*N*′-bis(2-pyridylmethyl)-*N*,*N*′-dimethylethane-1,2-diamine (BPMEN, **2**) [50] were synthesised in good yields following literature procedures (Supporting Information File 1). (+)-(2*R*,2′*R*)-1,1′-Bis(2-pyridylmethyl)-2,2′-bipyrrolidine ((*R*,*R*′)-PDP, **3**) was synthesised from commercially available (*R*,*R*′)-2,2′-bipyrrolidine L-tartrate trihydrate according to the procedure reported by White and Chen [51]. Ligands **1**–**3** were combined with iron(II) triflate using literature protocols to generate [Fe(TPA)(CH_3_CN)_2_](OTf)_2_ (FeTPA, **4**) [52], [Fe(BPMEN)(OTf)_2_] (FeBPMEN, **5**) [48] and [Fe(*R*,*R*′-PDP)(OTf)_2_] (Fe(*R*,*R*′)-PDP, **6**) (Supporting Information File 1) [51]. *N*-Boc-hydroxylamine (*tert*-butyl hydroxycarbamate, BocNHOH, **8**) was prepared using a modified literature procedure (Supporting Information File 1) [61].

### Hydroxyamination reactions

Acetonitrile was freshly distilled from calcium hydride; for reactions under argon (i.e., anaerobic conditions), the solvent was subjected to three freeze–thaw degassing cycles immediately before use. Stock solutions of iron complex (22.6 mmol L^−1^) and BocNHOH (**8**, 70 mmol L^−1^) in degassed acetonitrile were prepared under an atmosphere of argon. Acetonitrile (8.0 mL) was stirred under the required environment (argon or air) while iron complex stock solution (0.3 mL, 6.8 μmol) and cyclohexene (0.7 mL, 6.9 mmol) were added. Using a syringe pump, the BocNHOH stock solution (1.0 mL, 70 μmol) was added to the reaction mixture over 30 min. The reaction was stirred for 18 h after which time the solvent was removed in vacuo. The residue was dissolved in ethyl acetate and passed through a micro-column of silica to remove the iron complex. The sample was subjected to analysis by GC using *n*-decane as an internal standard and the single point internal standard method (Supporting Information File 1) [62–63]. Each reaction was performed in triplicate and data presented above are the average of the three runs.

## Supporting Information

Experimental procedures and characterization data for synthesis of ligands and iron complexes plus preparative-scale turnover reactions; details of GC conditions for analysis of turnover reactions; turnover data for control experiments and investigation of catalyst loading; UV–vis and ^1^H NMR spectra evincing the interaction of BocNHOH (**8**) with FeTPA (**4**).

File 1 Experimental procedures and characterization data, GC conditions, UV–vis and ^1^H NMR spectra.
