# Supplementary material for: Iron complexes of tetramine ligands catalyse allylic hydroxyamination via a nitroso–ene mechanism
Source: Beilstein J Org Chem. 2015 Dec 11;11:2549–56. doi: 10.3762/bjoc.11.275 (PMC4685922; doi:10.3762/bjoc.11.275)
Supplement: File 1 — Experimental procedures and characterization data, GC conditions, UV–vis and 1H NMR spectra. [file Beilstein_J_Org_Chem-11-2549-s001.pdf]

Supporting Information

for

Iron complexes of tetramine ligands catalyse allylic hydroxyamination via a nitroso–ene mechanism

David Porter, Belinda M.-L. Poon and Peter J. Rutledge*§

Address: School of Chemistry F11, The University of Sydney, NSW 2006, Australia

Email: Peter J. Rutledge - peter.rutledge@sydney.edu.au

* Corresponding author

§tel: +61 2 9351 5020; fax: +61 2 9351 3329

Experimental procedures and characterization data, GC conditions, UV–vis and ^{1}H NMR spectra

Contents

1. Synthesis of ligands **1–3**, iron complexes **4–6** and *N*-Boc-hydroxylamine (**8**) S1
2. Preparative-scale synthesis of reference compounds S4
3. GC conditions for analysis of turnover reactions S5
4. Turnover data for control experiments and investigation of catalyst loading S7
5. UV–vis and ^{1}H NMR spectra evincing the interaction of BocNHOH (**8**) with FeTPA (**4**) S8
6. References S9

1. Synthesis of ligands 1–3, iron complexes 4–6 and *N*-Boc-hydroxylamine (8)

*Tris(2-pyridylmethyl)amine (TPA, **1**)*

Prepared according to a literature procedure [1]. Pyridine-2-carboxaldehyde (0.50 mL, 10.5 mmol) was added to a mixture of 2-(aminomethyl)pyridine (0.27 mL, 2.59 mmol) and sodium triacetoxyborohydride (1.68 g, 7.96 mmol) in CH_2Cl_2 (25 mL) under an atmosphere of nitrogen with stirring. The mixture was stirred for a further 18 h at which point a saturated aqueous solution of $NaHCO_3$ solution was added. The mixture was extracted with ethyl acetate (3 × 20 mL) and the organic extracts dried over $MgSO_4$. Removal of the solvent gave an orange oil. Trituration with hexane gave the title compound as a yellow solid (0.61 g, 80%). Characterisation in agreement with

Britovsek et al. [1]. MS (ESI$^+$) *m/z*: 291 (75%, $[M+H]^+$); 313 (100%, $[M+Na]^+$); ^{1}H NMR (200 MHz, $CDCl_3$) δ_H: 3.89 (6H, s, 3 × CH_2); 7.14 (3H, m, 3 × pyr-CH); 7.56–7.70 (6H, m, 6 × pyr-CH); 8.53 (3H, m, 3 × pyr-CH).

*N,N′-Bis(2-pyridylmethyl)-N,N′-dimethylethane-1,2-diamine (BPMEN, **2**)*

Prepared according to a literature procedure [2]. Pyridine-2-carboxaldehyde (2.40 mL, 24.9 mmol) and ethane-1,2-diamine (0.83 mL, 12.5 mmol) were dissolved in methanol (20 mL) and heated at reflux under an atmosphere of nitrogen. After 2 h the reaction mixture was cooled to 0 °C and sodium borohydride (1.42 g, 37.5 mmol) was added. The mixture was then refluxed at 100 °C for 1 h and then cooled to room temperature. Hydrochloric acid (5 M) was added until the mixture reached pH 4; the resulting white precipitate was removed by filtration. The filtrate was treated with aqueous sodium hydroxide solution (2.5 M) to raise the pH to 9 and the resulting white precipitate removed by filtration. The solvent (methanol) was removed from the filtrate under reduced pressure and the residual aqueous phase extracted with chloroform (3 × 50 mL). The chloroform extracts were dried ($MgSO_4$) and the chloroform removed in vacuo to afford an orange oil. Without further purification, this oil was added to a mixture of formic acid (6.8 mL, 180 mmol), formaldehyde (5.6 mL, 187 mmol) and water (0.93 mL, 52 mmol). The mixture was heated at 85 °C for 3 days. The reaction mixture was cooled to room temperature and aqueous sodium hydroxide solution (2.5 mM) added until the pH was >12. The aqueous phase was extracted with chloroform (3 × 50 mL), the organic extracts dried ($MgSO_4$) and evaporated in vacuo to give **2** as a brown oil (2.60 g, 77%). Characterisation in agreement with Hureau et al. [2]. MS (ESI$^+$) *m/z*: 271 (100%, $[M+H]^+$); ^{1}H NMR (200 MHz, $CDCl_3$) δ_H: 2.27 (6H, s, 2 × CH_3); 2.65 (4H, s, CH_2CH_2); 3.68 (4H, s, 2 × pyr-CH_2); 7.14 (2H, app t, *J* = 6.0 Hz , 2 × pyr-CH); 7.41 (2H, d, *J* = 8.0 Hz , 2 × pyr-CH); 7.62 (2H, dt, *J* = 1.5, 8.0 Hz , 2 × pyr-CH); 8.53 (2H, app d, *J* = 5.0 Hz , 2 × pyr-CH).

*(+)-(2R,2′R)-1,1′-Bis(2-pyridylmethyl)-2,2′-bipyrrolidine ((R,R′)-PDP, **3**)*

(*R,R′*)-PDP was prepared according to literature procedure [3]. (*R,R′*)-2,2′-Bipyrrolidine L-tartrate trihydrate (0.50 g, 1.45 mmol) was dissolved in water (4 mL). Sodium hydroxide pellets were added (380 mg, 9.43 mmol) with stirring, followed by CH_2Cl_2 (5 mL). 2-Picolyl chloride hydrochloride (0.60 g, 3.66 mmol) was dissolved in water (1 mL) and added to the reaction mixture. After stirring for 18 h the reaction mixture was diluted with aqueous NaOH (2.5 M, 10 mL). The aqueous layer was extracted with CH_2Cl_2 (3 × 15 mL). The organic extracts were then dried ($MgSO_4$) and concentrated in vacuo. The crude product was purified by silica gel chromatography (ethyl acetate/hexane/triethylamine, 10:10:1) to give (*R,R′*)-PDP (0.41 g, 56%). Characterisation in agreement with White and Chen [3]. MS (ESI$^+$) *m/z*: 323 (100%, $[M+H]^+$); ^{1}H NMR (300 MHz,

$CDCl_3$) δ_H: 1.58–1.92 (8H, m); 2.24 (2H, app q; *J* = 8.5 Hz); 2.79 (2H, m); 3.00 (2H, p, *J* = 4.5 Hz); 3.51 (2H, d, *J* = 14.5 Hz); 4.20 (2H, d, *J* = 14.53 Hz); 7.11 (2H. app t, *J* = 6.0 Hz, 2 × pyr-CH); 7.40 (2H, d, *J* = 7.5 Hz, 2 × pyr-CH); 7.62 (2H, dt, *J* = 1.5, 7.5 Hz, 2 × pyr CH); 8.50 (2H, app d, *J* = 5.0 Hz, 2 × pyr-CH) $[\alpha]_D^{20}$**:** 155 (*c* 1.43, CH_2Cl_2); 126 (*c* 1.31, MeOH); (Lit.[3] $[\alpha]_D^{25}$: -94.6 (*c* 1.0 MeOH) for (*S,S′*)-**3**).

*[Fe(TPA)($CH_3CN)_2$]($SO_3CF_3)_2$ (FeTPA, **4**)*

FeTPA (**4**) was prepared according to a literature procedure [4]. TPA (**1**, 0.29 g 1.0 mmol) was dissolved in acetonitrile (4.0 mL) and then added to iron(II) triflate (0.44 g, 1.0 mmol). The reaction mixture was stirred for 30 min then cooled to −30 °C and treated with diethyl ether (4.0 mL) to precipitate the target compound as dark red crystals (0.40 g, 55%). Characterisation in agreement with Hagen and Diebold [4]. mp: 59–60 °C; MS (ESI$^+$) *m/z*: 495 (100%, [^{56}Fe(TPA)(OTf)]$^+$); 496 (24%, [^{57}Fe(TPA)(OTf)]$^+$).

*[Fe(BPMEN)($SO_3CF_3)_2$] (FeBPMEN, **5**)*

FeBPMEN (**5**) was prepared according to a literature procedure [1]. BPMEN (**2**, 0.31 g, 1.1 mmol) was dissolved in THF (30 mL) and added to iron(II) triflate (0.50 g, 1.1 mmol). The reaction mixture was stirred overnight and the solvent reduced in vacuo. The precipitate was collected by filtration and washed with THF (2 × 5 mL) to give **5** as a pale yellow solid (0.22 g, 30%). Characterisation in agreement with Britovsek et al. [1]. mp: 152–154 ^{o}C; MS (ESI$^+$) *m/z*: 475 (100%, [^{56}Fe(BPMEN)(OTf)]$^+$); 476 (24%, [^{57}Fe(BPMEN)(OTf)]$^+$); 478 (7%, [^{58}Fe(BPMEN)(OTf)]$^+$).

*[Fe^{II}(R,R′-PDP)($SO_3CF_3)_2$] (Fe(R,R′)-PDP, **6**)*

(*R*,*R′*)-PDP (**3**, 0.22 g 0.67 mmol) was dissolved in acetonitrile (4.0 mL) and added to iron(II) triflate (0.29 g, 0.67 mmol). The reaction mixture was stirred for 30 min then layered with diethyl ether at −30 °C. After standing for 30 min the ether layer was removed then treated with a CH_2Cl_2/hexane mixture, prompting precipitation of a yellow solid. The solvent was removed in vacuo and the yellow solid recrystallised three times from CH_2Cl_2/hexane to give the title complex **6** as a yellow solid (387 mg, 85%). mp: decomp. above 154 °C MS (ESI$^+$) *m/z*: 209 (100%; [^{56}Fe(*R*,*R′*-PDP)(CH_3CN)]$^{2+}$); 210 (27%, [^{57}Fe(*R*,*R′*-PDP)(CH_3CN)]$^{2+}$); 230 (25%, [^{56}Fe(*R*,*R′*-PDP)($CH_3CN)_2$]$^{2+}$); 527 (76%, ^{56}Fe(*R*,*R′*-PDP)(OTf)]$^+$); 528 (22%, ^{57}Fe(*R*,*R′*-PDP)(OTf)$^+$]).

*tert-Butyl hydroxycarbamate (BocNHOH, **8**)*

Synthesis of BocNHOH (**8**) was adapted from a literature procedure [5]. Hydroxylamine hydrochloride (0.54 g, 7.7 mmol) was dissolved in water (10 mL) at 0 °C. $NaHCO_3$ (0.65 g, 7.7

mmol) was added slowly portionwise while the temperature was maintained at 0 °C. Further $NaHCO_3$ (0.32 g, 3.8 mmol) was added and stirred to effect dissolution. A solution of di-*tert*-butyl dicarbonate (0.83 g, 3.8 mmol) in CH_2Cl_2 (10 mL) was added, and the biphasic system was stirred at 0 °C for 3 h, then at room temperature for 3 h. A saturated aqueous $NaHCO_3$ solution (3 mL) was added and the reaction mixture extracted with CH_2Cl_2 (3 × 20 mL). The organic extracts were combined, dried ($MgSO_4$) and the solvent reduced *in vacuo*. Precipitation with hexane at −20 °C yielded *tert*-butyl hydroxycarbamate **8** (0.46 g, 91%). Characterisation in agreement with Andrews et al. [6] mp: 47–49 °C (Lit. [7] mp: 54–56°C); ^{1}H NMR (200 MHz, d_6-DMSO) δ_H: 1.37 (9H, s, *t*-Bu); 8.46 (1H, s); 9.24 (1H, s).

2. Preparative-scale synthesis of reference compounds

*tert-Butyl cyclohex-2-enyl(hydroxy)carbamate (**9**)*

FeBPMEN (**5**, 22.4 mg, 35 µmol) and BocNHOH (**8**, 80 mg, 600 µmol) were dissolved in acetonitrile (35 mL). Cyclohexene (3.5 mL) was added and the reaction mixture stirred open to the atmosphere at room temperature for 18 h. The solvent volume was reduced in vacuo and the product was purified by column chromatography (silica gel, hexane/ethyl acetate 1:4) to give the title compound **9** (43 mg, 33%); R_f: 0.50 (hexane/ethyl acetate 1:4); MS (ESI^+) *m/z*: 214 (45%, $[M+H]^+$) ν_{max} ($CHCl_3$): 3687 (s, br), 3039 (m), 3016 (m), 1689 (s), 1365 (w); ^{1}H NMR (300 MHz, $CDCl_3$) δ_H: 1.48 (9H, s, $C(CH_3)_3$), 1.56–1.69 (1H, m, aliphatic C$\underline{H}_2$), 1.76–1.92 (3H, m, aliphatic C$\underline{H}_2$), 1.98–2.10 (2H, m, aliphatic C$\underline{H}_2$), 4.54 (1H, m, C$\underline{H}$N), 5.57 (1H, d, *J* = 10.0 Hz, C$\underline{H}$=CH) 5.90 (1H, app d, *J* = 10.0 Hz, CH=C$\underline{H}$), 6.60 (1H, br s, O$\underline{H}$); ^{13}C NMR (75 MHz, $CDCl_3$) δ_C: 21.2, 24.4, 25.6, 28.3, 55.6, 81.8, 126.9, 131.3, 157.0.

*4-Methyl-3,6-dihydro-[1,2]oxazine-2-carboxylic acid tert-butyl ester (**15**) and 5-methyl-3,6-dihydro-[1,2]oxazine-2-carboxylic acid tert-butyl ester (**16**)*

Synthesis of a 1:1 mixture of **15** and **16** was adapted from literature procedures [8,9]. Sodium periodate (44 mg, 0.20 mmol) was dissolved in an aqueous sodium acetate solution (0.5 M, 5 mL). The pH was adjusted to 6 with hydrochloric acid (5 M). Isoprene (**14**, 40 µL, 0.40 mmol) was dissolved in ethyl acetate (1 mL) and added to the aqueous phase. BocNHOH (**8**, 25 mg, 0.19 mmol) was dissolved in ethyl acetate (5 mL) and added to the reaction mixture. The reaction mixture was stirred at 0 °C for 1 h before addition of a saturated sodium thiosulphate solution (5 mL) and extraction with CH_2Cl_2 (3 × 10 mL). The organic extracts were dried ($MgSO_4$) and the solvent reduced in vacuo to yield the title compounds **15** and **16** as a 1:1 mixture (7 mg, 19%). Characterisation in agreement with Kouklovsky et al. [10]. MS (ESI^+) *m/z*: 222 (100%, $[M+Na]^+$); ^{1}H NMR (200 MHz, $CDCl_3$) δ_H: 1.49 (18H, s, $C(CH_3)_3$), 1.65 (3H, s, CH_3), 1.72 (3H, s, CH_3), 3.93

(2H, s, CH_2), 4.02 (2H, m, CH_2), 4.25 (2H, s, CH_2), 4.35 (2H, m, CH_2), 5.51 (1H, m, CH=C), 6.60 (1H, app s, CH=C).

*tert-Butyl hydroxy(2-methylenebut-3-enyl)carbamate (**17**)*

FeTPA (**4**, 10 mg, 14 μmol) and BocNHOH (**8**, 190 mg, 1.43 mmol) were dissolved in acetonitrile (50 mL). Isoprene (**14**, 300 μL) was added and the reaction mixture stirred for 18 h. The solvent was removed in vacuo and the product was purified by column chromatography (silica gel, hexane/ethyl acetate 4:1) to give the title compound **17** (22 mg, 8%); MS (ESI^+) *m/z*: 222 (80%, $[M+Na]^+$); ^{1}H NMR (300 MHz, $CDCl_3$) δ_H: 1.48 (9H, s, $C(CH_3)_3$), 4.27 (2H, s, CH_2N), 5.11 (1H, d, J = 11.0 Hz, C<u>H</u>=C), 5.20 (2H, app d, J = 5.0 Hz, CH_2=C), 5.25 (1H, d, J = 18.5 Hz, one of CH_2=C), 6.40 (1H, dd, J = 11.0, 18.5 Hz, one of CH_2=C), 6.59 (1H, br s, OH); ^{13}C NMR (75 MHz, $CDCl_3$) δ_C: 27.9 51.1, 81.8, 114, 117, 136, 140, 156.

3. GC conditions for analysis of turnover reactions

The extent of alkene turnover was determined using gas chromatography (GC). GC conditions were developed to achieve baseline separation of hydroxyamination product **9** from the internal standard *n*-decane and byproducts **10**–**12** (Table S1).

Table S1: Gradient GC elution conditions for acetamide determination.

Time (min)	Temp/Rate Change	Time	Final Temperature
0–2	70 °C	2 min	60 °C
2–13	12 °C/min	11 min	200 °C
13–15	No change	2 min	200 °C

Under these conditions, compounds **8**, **9**–**12** (cyclohexene turnover) and **15**–**17** (isoprene turnover) eluted with the retention times summarised in Table S2 (± 0.1 min), determined using authentic samples of each compound.

Table S2: Retention times for products of interest.

Compound	R_t (min)	Compound	t_R (min)
**8**	7.1	**12**	5.1
**9**	13.1	**15**/**16**	11.5/11.6
**10**	4.8	**17**	10.8
**11**	5.3	*n*-decane	6.8

The amount of each product was quantified using the single point internal standard method [11,12]. The internal response factor (IRF) for each product was calculated by preparing a solution containing an authentic sample of that product (2 mg) and *n*-decane standard (100 μL, 10 mg/mL)

in ethyl acetate. An aliquot of this solution (2 μL) was analysed by GC three times and the peak areas integrated. The IRF could then be calculated using this equation:

$$\text{IRF} = \frac{\text{area}_{\text{internal standard}} \times \text{amount}_{\text{specific compound}}}{\text{amount}_{\text{internal standard}} \times \text{area}_{\text{specific compound}}}$$

Then by adding a defined quantity (100 μL) of *n*-decane standard solution to the turnover reaction mixture immediately prior to GC analysis, the amount of product formed can then be calculated as:

$$\text{amount}_{\text{specific compound}} = \frac{\text{amount}_{\text{decane}} \times \text{area}_{\text{specific compound}} \times \text{IRF}}{\text{area}_{\text{decane}}}$$

4. Turnover data for control experiments and investigation of catalyst loading

Table S3: Control reactions for the catalytic allylic amination of cyclohexene (**7**); reaction conditions and analysis as for Scheme 1/Table 1.

**Entry** [a]	**Catalyst**	**mol %**	**Atmos.**	**9**	**10**	**11**	**12**
1	$Fe(OTf_2)$	10	Argon	1	0	0	3
2	**1**	10	Argon	4	5	3	4
3	**2**	10	Argon	7	2	2	3
4	none	-	Argon	0	2	1	3
5	none	-	Air	1	30	27	0

[a]Each reaction performed in triplicate; data are averages of at least three runs.

Table S4: The effect of catalyst loading on the allylic hydroxylamination of cyclohexene (**7**). Reaction conditions: catalyst **4** or **5** (1–20 mol %) and BocNHOH (**8**, 70 μmol) were dissolved in CH_3CN (9 mL) and stirred at room temperature under air while cyclohexene (0.7 mL, 7 mmol) was added, then overnight for 18 h.

**Entry** [a]	**Catalyst**	**mol %**	**9** [b,c]	**10** [b,c]	**11** [b,c]	**12** [b,c]
1	**4**	1	42	14	9	5
2	**4**	2	40	15	10	5
3	**4**	5	33	9	5	5
4	**4**	10	27	54	36	5
5	**4**	20	0	>100	>100	0
6	**5**	1	0	>100	>100	0
7	**5**	5	27	9	5	5
8	**5**	10	40	32	14	4
9	**5**	20	0	>100	>100	0

[a] Each reaction was performed in triplicate; data are averages of at least three runs.

[b] Yields determined by GC using single point internal standard method (vide supra).

[c] Yields are quoted relative to the initial amount of BocNHOH (**8**), limiting reagent for the hydroxyamination reaction of interest. Formation of products **10** and **11** is not dependent on hydroxylamine **8** so the combined yields for some entries in this table total >100%.

5. UV–vis and ^{1}H NMR spectra evincing the interaction of BocNHOH (8) with FeTPA (4)

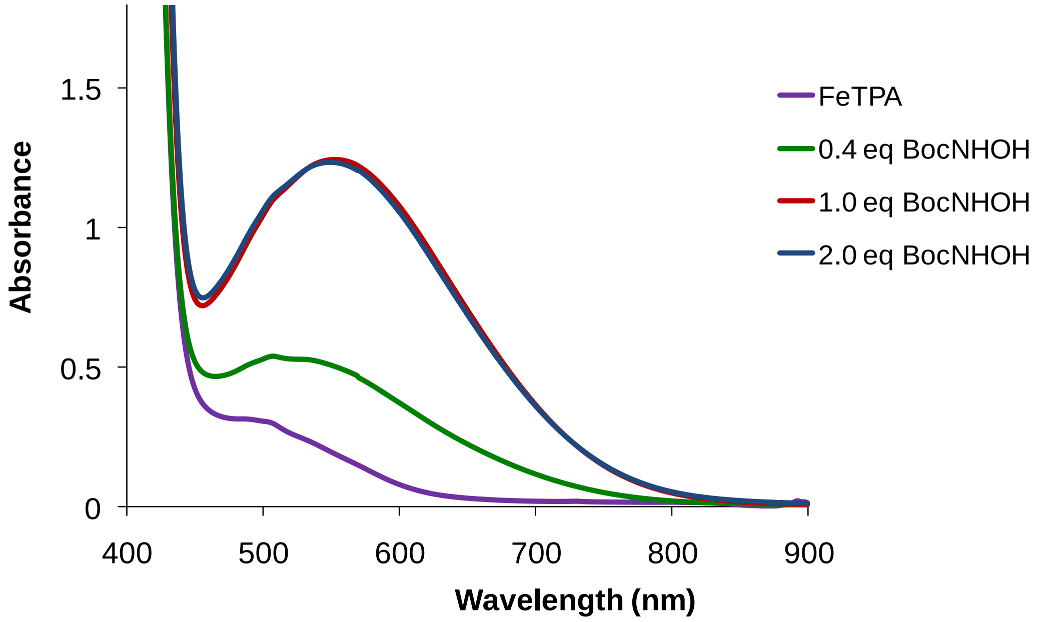

Figure S1: UV–vis titration of FeTPA with BocNHOH

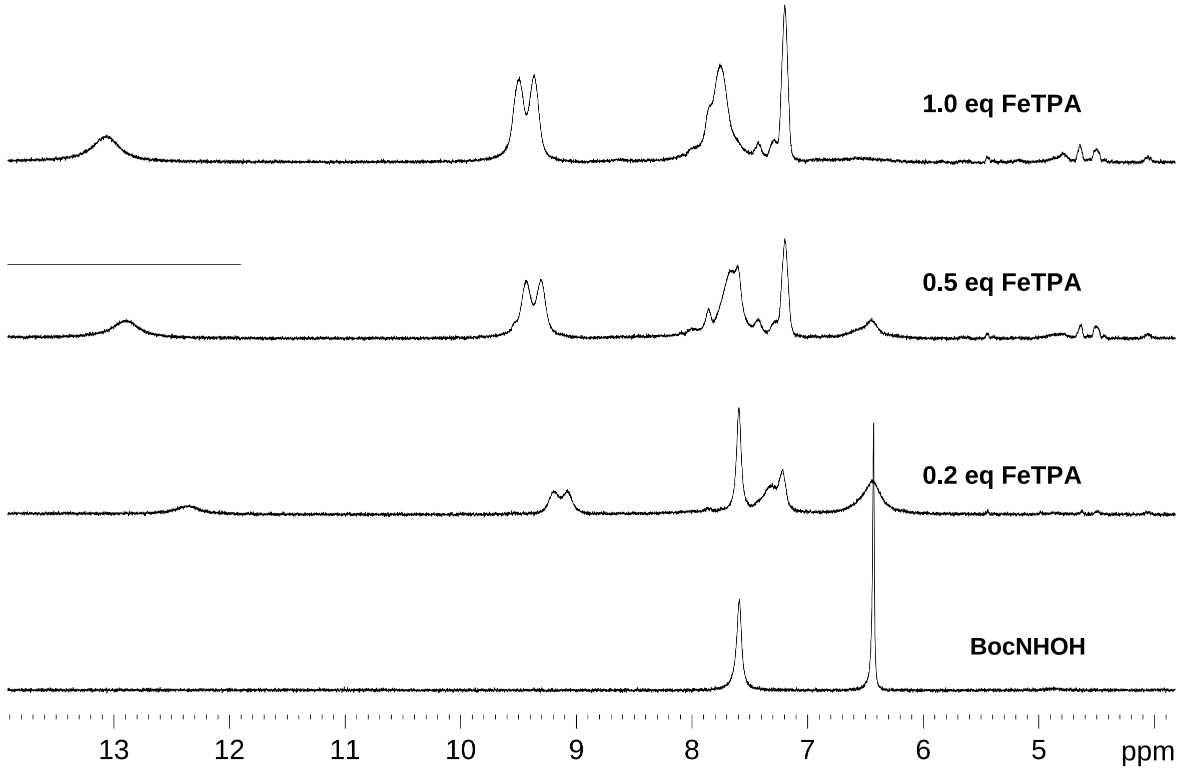

Figure S2: ^{1}H NMR titration of BocNHOH (**8**) with FeTPA (**4**) in d_3-acetonitrile: BocNHOH **8** (bottom) plus 0.2, 0.5 and 1.0 equivalents (top) of FeTPA (**4**). The hydroxylamine signals at 6.46 and 7.57 ppm (N–H and O–H protons) disappear as FeTPA is added. Once one equivalent of FeTPA has been added the *N*-Boc-hydroxylamine signal at 6.5 ppm is no longer present; the signal at 7.6 ppm is obscured by one of the pyridine signals from the TPA ligand, but the integration indicates that the hydroxylamine signal has also

disappeared once 1.0 equivalents of FeTPA have been added. This is consistent with coordination of hydroxylamine **8** to the FeTPA complex **4**.

6. References

1. Britovsek, G. J. P.; England, J.; White, A. J. P. *Inorg. Chem.* **2005**, *44*, 8125-8134.
2. Hureau, C.; Blondin, G.; Charlot, M. F.; Philouze, C.; Nierlich, M.; Cesario, M.; Anxolabehere-Mallart, E. *Inorg. Chem.* **2005**, *44*, 3669-3683.
3. Chen, M. S.; White, M. C. *Science* **2007**, *318*, 783-787.
4. Diebold, A.; Hagen, K. S. *Inorg. Chem.* **1998**, *37*, 215-223.
5. Cardillo, G.; Gentilucci, L.; Bastardas, I. R.; Tolomelli, A. *Tetrahedron* **1998**, *54*, 8217-8222.
6. Andrews, D. M.; Arnould, J. C.; Boutron, P.; Delouvrie, B.; Delvare, C.; Foote, K. M.; Hamon, A.; Harris, C. S.; Lambert-van der Brempt, C.; Lamorlette, M.; Matusiak, Z. M. *Tetrahedron* **2009**, *65*, 5805-5816.
7. Kalita, B.; Nicholas, K. M. *Tetrahedron Lett.* **2005**, *46*, 1451-1453.
8. Kirby, G. W.; Sweeny, J. G. *J. Chem. Soc. Perkin Trans. 1* **1981**, 3250-3254.
9. Kirby, G. W.; McGuigan, H.; Mackinnon, J. W. M.; McLean, D.; Sharma, R. P. *J. Chem. Soc. Perkin Trans. 1* **1985**, 1437-1442.
10. Calvet, G.; Blanchard, N.; Kouklovsky, C. *Synthesis-Stuttgart* **2005**, 3346-3354.
11. Loconto, P. R., *Trace Environmental Quantitative Analysis - Principles, Techniques and Applications*. Marcel Dekker Inc: New York, 2001; p 647.
12. Harris, D. C., *Quantitative Chemical Analysis, Sixth Edition*. W. H. Freeman: New York, 2003.
